# Case report: Exceptional transmission of congenital hyperinsulinism from a focal CHI mother to her diffuse CHI dichorionic diamniotic twins

**DOI:** 10.3389/fendo.2024.1408003

**Published:** 2024-06-17

**Authors:** Daniela Telehuz, Oana Plesa, Florence Bouilloud, Helene Wucher, Pascale De Lonlay, Claire-Marine Bérat, Cécile Saint-Martin, Olivier Dupuy, Jean-Baptiste Arnoux

**Affiliations:** ^1^ Groupe Hospitalier Paris Saint Joseph, Department of Diabetology, Endocrinology and Metabolic Diseases, Paris, France; ^2^ Hôpital Universitaire Necker - Enfants malades, Reference centre for inherited metabolic diseases, Assistance Publique–Hôpitaux de Paris (AP-HP), Paris, France Rare diseases network G2M, MetabERN, Paris Cité University, Paris, France; ^3^ Department of Medical Genetics, Assistance Publique–Hôpitaux de Paris (AP-HP) Pitié-Salpêtrière Hospital, Filière G2M, Assistance Publique–Hôpitaux de Paris (AP-HP), MetabERN, Paris, France

**Keywords:** ABCC8, pasireotide, diazoxide-unresponsive, compound heterotozygosity, congenital hyperinsulinism (CHI), congenital hyperinsulinaemic hypoglycaemia

## Abstract

We present the case of a 36-year-old female who was diagnosed at birth with CHI that caused severe hypoglycaemia unresponsive to Diazoxide. Subtotal pancreatectomy was performed at the age of three weeks. Later, histological analysis of her pancreas in a research setting revealed a focal form of CHI. Genetic testing was not available at that time. The patient developed pancreatic exocrine deficiency and insulin-dependent diabetes at the age of 9 years. In 2016, a genetic test revealed a missense heterozygous variant in the ABCC8 gene inherited from her father and classified as having a recessive inheritance. The geneticist concluded that the risk of CHI for her offspring would be low (1/600), making pregnancy favourable. As there was no consanguinity in the family, testing the future father was deemed unnecessary (carrier frequency 1/150 in the general population). The pregnancy occurred spontaneously in 2020 and at a gestational age of 28 weeks, the mother went into premature labour. An emergency C-section was performed in April 2021 resulting in the birth of bichorial bi-amniotic male twins. Following birth, both newborns experienced persistent severe hypoglycaemia which required glucagon treatment and intravenous glucose infusion initially, followed by Diazoxide from day 51 after birth, without satisfactory response. Continuous intravenous Octreotide treatment was introduced on day 72. Due to the recurrence of hypoglycaemia episodes despite reaching maximum doses of Octreotide, from day 92 the treatment was switched to Pasireotide. Genetic tests revealed the same genotypes for both infants: the exon 39 missense variant (c.4716C>A; p.Ser1572Arg) inherited from their mother and a truncating variant in exon 28 (c.3550del; p.Val1184*), inherited from their asymptomatic father. As a result of inheriting two recessive variants of the ABCC8 gene, the children were diagnosed with a diffuse form of CHI, consistent with the diazoxide-unresponsive presentation. This situation is very rare outside consanguinity. This case emphasises the significance of genetic counselling for individuals with a history of rare diseases outside the context of consanguinity, as there is a potential risk of recurrence. Prenatal diagnosis can lead to better outcomes for affected neonates, as well as help families make informed decisions about future pregnancies.

## Introduction

Congenital hyperinsulinism (CHI) is a rare but severe cause of hypoglycaemia at birth and during infancy, characterised by abnormal insulin secretion (1). While non-genetic forms of HI in neonates are transient, caused mostly by perinatal stress, or a mother’s uncontrolled diabetes, CHI is monogenic and can present either as an isolated CHI due to a variant in a gene directly involved in insulin secretion or as a syndromic CHI, such as Kabuki, Turner or Beckwith-Wiedemann syndromes.

To date, ten genes have been linked to isolated CHI, explaining 45–55% of the cases (*ABCC8, KCNJ11, GLUD1, GCK, HADH, HNF1A, HNF4A, SLC16A1, UCP2, HK1*) and more than 28 genes or chromosomal abnormalities have been reported for syndromic CHI. Most patients carry variants in *ABCC8* and *KCNJ11* variants that cause an adenosine triphosphate-sensitive potassium channels (KATP channel) defect, leading to dysregulated insulin secretion (2).

The *ABCC8* gene is located on the chromosomal region 11p15.1 and encodes the sulphonylurea receptor 1 (SUR1) subunit of the KATP channel. This gene is responsible for some of the most severe CHIs that are unresponsive to medical therapies.

KATP-CHI can lead to two different histological forms: a focal form which is characterised by a localised pancreatic hyperplasia of beta-cells that can be cured surgically, and a diffuse form, which is more severe, requiring long-term medication or, when unresponsive, a subtotal pancreatectomy having only a palliative purpose (3).

Focal forms of CHI are always linked with a paternally inherited recessive variant in the *ABCC8* or *KCNJ11* genes. The occurrence of a focal form requires a second hit, which is a uniparental disomy of the 11p15 chromosomal region of paternal origin and is limited to the focal lesion (4). On the other hand, the diffuse forms of CHI are associated with genotypes that involve either the presence of a dominant variant of the *ABCC8* gene or compound heterozygous or homozygous recessive variants of *ABCC8* (5).

There may be no difference in phenotypes between neonates with a focal form of CHI and those with a diffuse form of CHI, whether the latter is due to a dominant variant in *ABCC8*, or due to recessive heterozygous or homozygous variants in *ABCC8* (6).

In this article, we present the cases of three patients, a mother and her male twins, who were diagnosed with two different forms of CHI.

## Case presentation

We present the case of a 36-year-old female born in 1985 to non-consanguineous French parents. She was diagnosed with neonatal congenital hyperinsulinism in the context of severe hypoglycaemia that was unresponsive to Diazoxide treatment.

A subtotal pancreatectomy was performed at the age of three weeks, which cured hypoglycaemia. That was the current standard of care at that time because the possibility of focal forms was not yet known (7). At the age of two years, a histological analysis of her pancreas in a research setting showed a focal lesion (localised adenomatoid hyperplasia of beta-cells). Genetic testing was not available at that time. The patient developed a pancreatic exocrine deficiency and insulin-dependent diabetes at the age of 9 years and is currently being treated by an insulin pump.

In 2016, the patient was planning a pregnancy therefore her endocrinologist referred her to a geneticist doctor for counselling. Considering the patient’s history, a genetic test was performed that evidenced a missense heterozygous variant in the ABCC8 gene (gene nomenclature: exon 39 NM_001287174.3:c.4716C>A, protein nomenclature: p.Ser1572Arg) inherited from her father. This variant has never been reported in the literature before 2020 (8). Given the focal form at the pancreatic histology, the variant found in the *ABCC8* gene was classified as having a recessive inheritance. The geneticist concluded that the risk for her offspring to have CHI was very low (1/600) (9) and was favourable for the pregnancy. As there was no consanguinity in her couple, testing the future father was deemed unnecessary.

In the year 2020, a spontaneous dichorionic diamniotic pregnancy was initiated. In 2021, at a gestational age of 26 weeks and 5 days, the mother went into premature labour due to premature rupture of membranes. An emergency C-section was performed at 28 weeks and 6 days due to foetal arrhythmia, considered a sign of foetal distress. This led to the birth of male twins, with one weighting 2094 g (the first newborn), having an APGAR score of 7/8/10 and the other weighting 2020 g (the second newborn) and having an APGAR score of 2/4/6/7. Both children had respiratory distress syndrome, but only the second newborn needed surfactant administration and high-frequency oscillatory ventilation. Additionally, both children experienced severe persistent hypoglycaemia, requiring prolonged hospitalisation. Consequently, the intravenous glucose infusion rate was augmented up to 13.9 mg/Kg/min (maximum carbohydrate intake of 20g/Kg/day). Because the glucose infusion rate required to avoid hypoglycaemia was very high, there was a risk of fluid overload. To limit this risk, a continuous infusion of glucagon (cumulated dose of 1 mg per day) was started from the first day of life and was continued for approximately two months (it was stopped on day 62 for the first newborn, while the second newborn still needed intermittent use of Glucagon during the third and fourth months of life). On the ninth day (at 30 weeks and 1 day), the blood tests indicated hyperinsulinism for both newborns. The first newborn had a blood glucose level of 2,9 mmol/L (52 mg/dL), insulin 66 mUI/L, GH 42,9 mUI/L, IGF1 52 ng/mL and cortisol 241 nmol/L. The second newborn had a blood glucose level of 2,9 mmol/L (52 mg/dl), GH 42,30 mUI/L, IGF1 37 ng/mL, insulin 73 mUI/L, c-peptide 6,5 µg/L, cortisol 197 nmol/L. Transient HI was initially the main hypothesis because of several risk factors for it: mild perinatal stress, prematurity and maternal diabetes.

Because of prematurity and to limit the risk of complications, Diazoxide treatment was introduced only on day 51 at a dose of 5mg/Kg/day and was progressively increased to 10 mg/Kg/day. However, it did not result in any notable improvement and was discontinued respectively on day 69 (for the second child) and on day 72 (for the first child), due to diazoxide-associated pulmonary hypertension and acute pulmonary oedema, which both infants developed with a time difference of three days. Following this, non-invasive ventilation and diuretics were administered. On day 72, continuous intravenous octreotide treatment was introduced and its dose was gradually increased until reaching 20 µg/kg/day. The route of administration was then changed to a subcutaneous pump, achieving levels of 50 µg/kg/day for the first infant and 38 µg/kg/day for the second infant. Although well-tolerated, the persistence of hypoglycaemic episodes despite maximum doses of Octreotide led to the switch of this treatment to a different Somatostatin analogue expected to have a greater hyperglycaemic effect, Pasireotide, which was administered through a continuous subcutaneous pump at a rate of 1000 µg/m^2^/day from day 92 onwards. Despite this aggressive treatment regimen, there were still a few episodes of hypoglycaemia, which necessitated the augmentation of the dose to 1070 µg/m^2^/day for the first infant and 1200 µg/m^2^/day for the second infant, respectively.

## Genetic testing

Genetic testing was performed and it revealed that both infants had the same genotype: they inherited the exon 39 missense variant (c.4716C>A; p.Ser1572Arg) from their mother and a truncating variant in exon 28 (c.3550del; p.Val1184*) from their asymptomatic father. Due to the compound heterozygosity of two recessive variants, the children were diagnosed with a diffuse form of congenital hyperinsulinism.

## Outcome and follow-up

At the age of five months, the twins were discharged from the hospital and received continuous enteral nutrition at home, gradually reducing their carbohydrate intake. They also continued their treatment with Pasireotide at home through a continuous subcutaneous pump, which was initially managed by a specially trained nurse at home, then by the parents. At the age of 12 months, subcutaneous Pasireotide was switched to the long-acting form (one injection every 4 weeks), and Diazoxide treatment was started again and is well tolerated until now. Hypoglycaemic episodes are rare under this heavy treatment, and therefore, the children were not considered for subtotal pancreatectomy.

At the age of 3, both twins are still under heavy treatment, including Diazoxide 25 mg TID, an intramuscular long-acting Pasireotide injection of 20 mg every 4 weeks, and continuous enteral feeding at night (glucose rate 3.5 mg/Kg/min) and bolus/meals + raw corn starch every 4h during day time. Fortunately, they are both achieving the developmental milestones and normal growth: 17.1 Kg (+2.2 SD), 95.8 cm (+0.6 SD) for the first twin and 16.5 Kg (+1.7 SD), 95.2 cm (+0.5 SD) for the second twin. They will start normal schooling in a few months. During the follow-up, they did not experience any side effects of their treatment, except the usual Diazoxide-induced mild hyperpilosity.

Regarding the status of recessive variant carriers on *ABCC8* of both parents, the couple was informed of the high risk of recurrence of CHI for each new pregnancy: 25% risk for a diffuse form, but also an estimated 1/1200 risk for a focal form (1/2 risk for the father to transmit his variant and 1/600 risk to develop a 11p15 uniparental disomy in the pancreas). Due to the severity of the disease in this family, a prenatal diagnosis would be proposed to the couple.

## Discussion

Congenital hyperinsulinism is a common cause of persistent hypoglycaemia in newborns and infants, characterised by an abnormal secretion of insulin by the pancreatic beta-cells. Despite significant progress in understanding its pathophysiology and easing the diagnosis process considerably, managing the disease remains a challenge for paediatricians. Preventing hypoglycaemic episodes is essential in avoiding neurological damage which is the main complication of this disease (10).

During hypoglycaemia, inappropriate levels of insulin and C-peptide result in the inhibition of lipolysis, gluconeogenesis and ketogenesis, leading to a reduction of the brain’s alternative energy sources. This, in turn, increases the risk of hypoglycaemic-induced brain injury which can manifest through epilepsy, cognitive impairment, or other forms of neurological damage, if not treated urgently (3).

In physiological conditions, the secretion of insulin is triggered by a surge in blood glucose levels, exceeding a threshold of approximately 80 mg/dL. This results in glucose entering the pancreatic beta-cell via glucose transporters and undergoing aerobic oxidation in the mitochondria, thereby generating ATP. This triggers the closure of the KATP channel, leading to membrane depolarisation and the subsequent activation of voltage-dependent calcium channels. This, in turn, leads to an elevation in cytosolic calcium levels, facilitating insulin secretion.

Monogenic mutations in the genes encoding the KATP channel lead to a dysregulation of insulin secretion, resulting in abnormal levels of insulin and C-peptide. In particular, unsuppressed insulin and c-peptide levels are considered abnormal in the presence of low blood glucose <50 mg/dL. The *ABCC8* gene, located on the chromosomal location 11p15.1, encodes the SUR1 subunit of the KATP channel that is located in the pancreatic beta cells (11). The KATP channel comprises four SUR1 subunits, which are responsible for regulating the activity of the Kir6.2 protein that creates a pore on the beta cell membrane for the potassium influx. The closure of the KATP channels leads to membrane depolarisation, voltage-dependent calcium channel activation and subsequent insulin exocytosis (6). SUR1 is also the binding site for sulphonylureas such as diazoxide. Mutations in *ABCC8* are the most frequent cause of diazoxide-resistant CHI (12).

Our case report focuses on a female patient for whom the precise diagnosis of her neonatal CHI was confirmed through genetic testing more than two decades after the onset of her initial disease. The test revealed a missense heterozygous variant in the *ABCC8* gene inherited from her father, which has never been reported in the literature before 2020 (8). Given the focal form at the pancreatic histology, the variant found in the ABCC8 gene was classified as having a recessive inheritance. Indeed, dominant variants can only cause diffuse forms of CHI, while focal forms are associated with the paternally inherited recessive variant in the *ABCC8* gene. Thus, the apparition of a focal form requires a second hit, a uniparental disomy of the 11p15 chromosomal region of paternal origin, restricted to the focal lesion (4).

The patient was reassured regarding the low statistical risk of CHI in her children (1/600) (9), as her *ABCC8* variant had a recessive mechanism, would be maternally transmitted and there would be a low risk for her husband to be a carrier of a recessive variant in *ABCC8*. However, unfortunately, her twins were born with severe diffuse CHI due to the transmission of her ABCC8 pathogenic variant and an unexpected variant from her asymptomatic husband. The paternal variant was not previously described to our knowledge. To date, it is absent from the Human Gene Mutation Database (HGMD).

Diazoxide is the first-line treatment of CHI and it binds directly to the SUR-1 subunit to open the potassium channel of the pancreatic beta cell, thus inhibiting insulin secretion. In clinical practice, it is important to assess the diazoxide response of infants, as soon as possible to understand the severity of the disease and organise the best care for the child (13). In case of unresponsiveness after 5 days of Diazoxide treatment, a genetic analysis of ABCC8 and KCNJ11 genes is recommended (14). Suppose the genetic result is not directly diagnostic for a diffuse form (e.g. two recessive variants, or one dominant variant). In that case, the patient will be screened for a focal form with a pancreatic 18F-dihydroxyphenylalanine (DOPA) PET scan. In case of a focal lesion localised by the PET scan, a limited pancreatic resection can eventually cure the disease (13).

In our case, an identical genotype was found in both infants: two pathogenic recessive variants in ABCC8. This compound heterozygosity sets the diagnosis of a diffuse form of congenital hyperinsulinism, consistent with the diazoxide-unresponsive presentation of the children. Therefore, a pancreatic 18F-dihydroxyphenylalanine (DOPA) PET scan was unnecessary, as it is generally needed for localising focal forms of CHI (14).

Somatostatin synthetic analogues (SSAs) are the second line of therapy for CHI patients when they don’t respond to Diazoxide or when its use is contraindicated (15). These molecules bind to the somatostatin receptors (SSTR) and decrease the activity of the insulin gene promoter, thus reducing insulin biosynthesis and secretion (3, 16). The administration of SSA through subcutaneous injections suppresses insulin secretion, thus maintaining normal blood glucose levels (16). In CHI, SSA treatment usually starts with low doses of fast-acting analogues, progressively increasing to test their efficacity. After a few months, fast-acting SSAs can be switched to long-acting SSAs as a long-term therapy (13). The global evolution of CHI is towards a spontaneous progressive improvement of the disease (17).

In our case, the male twins were treated initially with Diazoxide, which didn’t have the expected effects on blood glucose levels. Moreover, it caused hemodynamic complications. As a result, we had to stop using Diazoxide. After that, we used short-acting continuous Octreotide as a second-line treatment. The doses are typically prescribed in a range of 5 to 40 micrograms per kilogram per day. However, it should be noted that treatment supplementary effects are rarely observed beyond 20 micrograms per kilogram per day in the majority of cases, despite reports of higher dosages being used (5). In our case, because Octreotide was well tolerated, we continued to increase the dosage gradually until the first infant reached 50 µg/kg/day and the second infant reached 38 µg/kg/day. When considering the use of SSAs in the treatment of CHI, it is essential to account for the possibility of tachyphylaxis. This can occur within 48 hours of initiating treatment (18).

In our case, the lack of efficiency despite the growing doses prompted us to discontinue the treatment and start administering a different SSA, Pasireotide, which exhibits moderate affinity towards SSTR2 and SSTR3 (19). It also demonstrates superior affinity towards SSTR5 compared to Octreotide. This has been found to cause less inhibition of glucagon secretion than Octreotide, thereby conferring advantages in the counterregulatory response to hypoglycaemia in CHI patients (20). SSTR5 and SSTR2 are the most expressed SSTR in the human ß-cells. Additionally, SSTR5 is the primary SSTR in the L-cells of de duodenum, which are endocrine cells that produce GLP-1. The latter potentiates glucose-stimulated insulin secretion in ß-cells (21, 22). As a result, Pasireotide can inhibit insulin secretion through its action on the SSTR5, either directly (through SSTR5 expressed by the ß-cells), or indirectly (through SSTR5 expressed by the L-cells). Consequently, in our case, Pasireotide was expected to have a more significant inhibiting effect on insulin secretion than Octreotide.

The most severe patients with diffuse forms of CHI might be considered for near-total pancreatectomy. However, this surgery should be avoided whenever possible, because it will lead to insulin-requiring diabetes starting from childhood, without a clear preventing effect on the cognitive outcome. Thus, there is still a great need to find more effective anti-hypoglycaemic medications for patients with severe CHI, and several research projects are ongoing (23, 24). Pluripotent stem cell-derived islets might help develop more efficient medications, thus avoiding a near-total pancreatectomy (25).

## Conclusion

Neonatal hyperinsulinism is a rare but serious condition that can cause severe hypoglycaemia and long-term neurological damage if not treated promptly (26, 27). While consanguinity is a well-known risk factor for this condition, there is evidence suggesting that it can also occur in non-consanguineous families due to *de novo* mutations (28).

In the presented case, the mother was found to be carrying a recessive mutation of the ABCC8 gene, whereas the father was an asymptomatic carrier. This particular scenario, observed outside of consanguinity, represents a rare event and poses a significant challenge for the medical community, as it prompts us to reevaluate the context of genetic testing beyond consanguinity and raises pertinent questions regarding its implementation.

As such, early detection through genetic testing (29) can lead to better outcomes for affected neonates, as well as help families make informed decisions about future pregnancies. It is imperative to consider genetic testing as a tool for the prevention and management of neonatal hyperinsulinism outside the context of consanguinity when one of the parents is an already known carrier of an ABCC8 mutation. By doing so, healthcare providers can provide optimal care to affected neonates and their families, while minimising the risk of recurrence and long-term complications.

## Data availability statement

The original contributions presented in the study are included in the article/supplementary material, further inquiries can be directed to the corresponding author/s.

## Ethics statement

Written informed consent was obtained from the individual(s), and minor(s)’ legal guardian/next of kin, for the publication of any potentially identifiable images or data included in this article.

## Author contributions

DT: Writing – original draft, Writing – review & editing. OP: Writing – review & editing. FB: Resources, Writing – review & editing. HW: Resources, Writing – review & editing. PD: Writing – review & editing. C-MB: Writing – review & editing. CS-M: Writing – review & editing. OD: Writing – review & editing. J-BA: Supervision, Validation, Writing – review & editing.
